# Genomics and metagenomics of *Madurella mycetomatis*, a causative agent of black grain mycetoma in Sudan

**DOI:** 10.1371/journal.pntd.0010787

**Published:** 2022-11-02

**Authors:** Anastasia P. Litvintseva, Sahar Bakhiet, Lalitha Gade, Darlene D. Wagner, Ujwal R. Bagal, Dhwani Batra, Emily Norris, Lavanya Rishishwar, Karlyn D. Beer, Emmanuel Edwar Siddig, Najwa Adam Mhmoud, Nancy A. Chow, Ahmed Fahal

**Affiliations:** 1 Mycotic Diseases Branch, Centers for Disease Control and Prevention, Atlanta, Georgia, United States of America; 2 The Mycetoma Research Centre, University of Khartoum, Khartoum, Sudan; 3 IHRC Inc., Atlanta, Georgia, United States of America; 4 ASRT Inc. Atlanta, Georgia, United States of America; 5 Office of Advanced Molecular Detection, Centers for Disease Control and Prevention, Atlanta, Georgia, United States of America; 6 Applied Bioinformatics Laboratory, Atlanta, Georgia, United States of America; 7 School of Biological Sciences, Georgia Institute of Technology, Atlanta, Georgia, United States of America; Tufts Medical Center, UNITED STATES

## Abstract

*Madurella mycetomatis* is one of the main causative agents of mycetoma, a debilitating neglected tropical disease. Improved understanding of the genomic diversity of the fungal and bacterial causes of mycetoma is essential to advances in diagnosis and treatment. Here, we describe a high-quality genome assembly of *M*. *mycetomatis* and results of the whole genome sequence analysis of 26 isolates from Sudan. We demonstrate evidence of at least seven genetically diverse lineages and extreme clonality among isolates within these lineages. We also performed shotgun metagenomic analysis of DNA extracted from mycetoma grains and showed that *M*. *mycetomatis* reads were detected in all sequenced samples with the average of 11,317 reads (s.d. +/- 21,269) per sample. In addition, 10 (12%) of the 81 tested grain samples contained bacterial reads including *Streptococcus* sp., *Staphylococcus* sp. and others.

## Author summary

Mycetoma is a debilitating disease recognized as a neglected tropical disease by the World Health Organization. The etiology of mycetoma is poorly understood; approximately 60% of cases are caused by fungi and the rest are bacterial, although this varies by region. The pathogenic fungus, *Madurella mycetomatis*, is most frequently identified from mycetoma cases. Here, we present a high-quality genome assembly of *M*. *mycetomatis* and results of the whole genome sequence analysis of 26 isolates from Sudan. We demonstrate evidence of at least seven genetically diverse lineages and extreme clonality among isolates within these lineages. Shotgun metagenomic analysis of DNA from mycetoma grains confirmed that *M*. *mycetomatis* was the predominant causative agent of eumycetoma Sudan; however, 10% of grains also contained bacterial reads. Thorough understanding of the genetic structure and diversity of fungi causing mycetoma is essential for the development of new diagnostic methods and identifying potential drug targets.

## Introduction

Mycetoma is a devasting, neglected chronic inflammatory disease [1,2] endemic to many tropical and subtropical regions of the world [3,4]. More than 70 bacterial and fungal organisms are reported to cause mycetoma [5–7]. The prevalence of eumycetoma (caused by fungi) and actinomycetoma (caused by bacteria) varies in different geographic regions, and the epidemiology of this disease is poorly understood.

Despite the wide diversity of causative agents, clinical presentation of mycetoma is often similar [6]. Patients usually present with a painless soft tissue swelling with multiple sinuses secreting sero-purulent discharge containing grains, which are compact masses of fungal hyphae or bacteria encapsulated in human tissues [6,8]. The inflammatory process usually starts in the subcutaneous tissue, often after a traumatic inoculation of the causative organisms and ultimately spreads to the deep tissues and bone. Eventually, this leads to massive tissue destruction, deformities, disabilities, and stigma and embarrassment for patients and families [6,9]. Although any body part can be affected, the extremities, especially the foot and hand, are most frequently involved.

Current mycetoma treatment modalities are limited, invasive, and have many adverse side effects [10]. Treatment outcome is characterized by low cure rates, high follow-up drop-out rates among patients, and high recurrence rates [6]. Eumycetoma is treated with a combination of pharmaceutical treatment and surgical excision, while actinomycetoma cases respond to pharmaceutical treatment alone [10,11]. Identifying the organism to the species level is mandatory to establish a proper diagnosis of mycetoma and advise the appropriate treatment modality [1,12]. Numerous tests and techniques are required, including surgical biopsy for tissue histopathological examination and grain extraction for culture and molecular diagnosis. The currently available diagnostic tools are of low sensitivity and specificity, invasive, expensive, have many side effects, and are not accessible or available in endemic regions [13].

In Sudan, eumycetoma accounts for 70% of mycetoma cases, most of which are caused by *Madurella mycetomatis* [7]; however, other fungi and bacteria were identified using molecular techniques [14,15]. The effectiveness of antifungal treatment for eumycetoma is not well established. Many patients continue antifungal treatment for prolonged periods with no clinical improvement or cure. Furthermore, most of the available antifungal drugs are toxic and have severe side effects. Hence, it is vital to identify the eumycetoma causative microorganisms and determine its susceptibility to various treatment options to avoid complications and side effects. With this background, the present study set out to better understand the causative agents of eumycetoma in Sudan and to assess the genomic diversity among *M*. *mycetomatis* isolates by whole genome sequencing to provide a framework for the development of molecular diagnostic methods for mycetoma.

## Materials and methods

### Ethics statement

This study was approved by the Mycetoma Research Centre Institutional Review Board (IRB) No. 345/2019. Every patient/guardian gave a written informed consent for using remnant clinical samples for research purposes.

### Patients and specimens

Patients for this study were diagnosed at the Mycetoma Research Center (MRC), University of Khartoum, Khartoum, Sudan and were enrolled in this study based on availability. Two convenience samples were collected: isolates for whole genome sequencing (WGS) were from one group of patients, while mycetoma grains used for DNA shotgun metagenomics were obtained from a different group of patients. While basic demographic information, including geographic origin, sex, age and occupation was available for patients from whom isolates were received, no information was available for patients from whom grains used for metagenomic analysis were obtained.

### Sample collection and processing

Grains from 178 patients were collected by deep surgical biopsy as part of routine clinical care. Grains were classified according to their color into black, yellow, and white grains. When applicable, the biopsy material was divided into three parts for histopathology, culture and DNA extraction. The part selected for histopathology was immediately immersed in 10% neutral buffer formalin and sent to the histopathology laboratory, where it was processed. The other two parts were immersed in sterile normal saline and sent to the MRC mycology laboratory for DNA extraction and culture.

### Histopathology

Histopathological examination was used to confirm the diagnosis of eumycetoma. Formalin blocks were prepared, and from each block, a single section was cut using a rotary microtome (Leica, Germany) at 3–5 μm thickness. Sections were then stained using Hematoxylin and Eosin. Grains containing characteristic fungal elements were classified as eumycetoma.

### Culture

Grains were washed three to four times in sterile saline, inoculated in Sabouraud dextrose agar (Oxoid, ThermoFisher, Waltham, MA USA) and incubated at 37°C for 1–6 weeks. When fungal growth was observed on the plate, it was identified based on its macroscopic and microscopic morphology. DNA from isolates was extracted using the DNeasy Blood and Tissue kit (Qiagen, Gaithersburg, MD, USA) according to the manufacturer’s instructions.

### DNA extraction from grain

Grains from 128 patients were transferred to a sterile Eppendorf tube containing 10 metal beads and 700 μl Bashing bead buffer (Zymo DNA fungal/bacteria extraction kits, Irvine, CA, USA) for DNA extraction. The mixture was then lysed using a Tissuelyser II (Qiagen, Germany) for 3 minutes at 30 hrz. The supernatant was removed, added onto a Zymo-Spin III-F filter column and processed following the manufacturer’s instructions. The DNA was extracted within 5 hours after the biopsy samples were collected. The obtained DNA was stored at -20°C and sent to CDC for further analysis. Upon arrival, the quality and quantity of DNA was evaluated by spectrophotometer and 81 samples with concentrations above 40 ng/μl and the ratio of absorbance at 260 nm and 280 nm between 1.8 and 2 were selected for sequencing.

### Short read sequencing

DNA from grains and *M*. *mycetomatis* isolates was sequenced using Illumina platform. Genomic libraries were constructed and barcoded using the NEB Next Ultra DNA Library Prep kit for Illumina (New England Biolabs, Ipswich, MA, USA) following manufacturer’s instructions. Libraries were sequenced on either the Illumina HiSeq 2500 platform (Illumina, San Diego, CA, USA) using the HiSeq Rapid SBS Kit v2 500-cycles or the MiSeq platform using the MiSeq Reagent Kit v2 500-cycles or Illumina MiSeq Reagent Kit v3 (600- cycles). Both platforms generated 251 bp paired reads.

### Long read sequencing

Mmyc_Sud5 isolate from a patient from El Gaziera State was selected for long read sequencing using PacBio RS II Sequel system sequencing system (Pacific Biosciences, Menlo Park, CA, USA) to improve assembly. This isolate was selected because most patients in our study were from this state. Twenty kb libraries were generated with the SMRTbell template prep kit 1.0 (Pacific Biosciences). Libraries were bound to polymerase using the DNA/polymerase binding kit P6 v2 (Pacific Biosciences), loaded on two SMRT cells (Pacific Biosciences), and sequenced with C4 v2 chemistry (Pacific Biosciences).

### Genome analysis

PacBio longreads obtained from Sequel were first trimmed to remove the barcode and then assembled using HGAP 4 [GenomeLength = 37000000] from Smrtlink v8.0.0.79519 (Pacific Biosciences, Menlo Park, CA, USA). The above genome assembly was used as a reference genome to identify single nucleotide polymorphisms (SNPs) using whole genome sequencing, indels were excluded. Whole genome SNP analysis was performed using MycoSNP (v0.21) analytical workflow (https://github.com/CDCgov/mycosnp; [16]) using the PacBio sequence of isolate Mmyc_Sud5 as the reference. Briefly, around 8.2% of the reference genome was masked for repetitive elements using MuMmer v/4.0. The raw illumina reads were trimmed using FaQCs version 2.10 [17]. Trimmed reads were aligned using the Burrows Wheeler Alignment (BWA mem) algorithm version 0.7.17 [18]. The resultant bam files were further processed by Samtools version 1.10 and Picards version 2.22.7 (http://broadinstitute.github.io/picard/) for variant calling using GATK version 4.1.4.1 [19]. Along with using a conditional variant filtration expression (QD < 2.0 || FS > 60.0 || MQ < 40.0), customized filtering criteria (a minimum GQ of 50, a minimum of 80 percent support to the alternate allele in AD, and a minimum DP of 10) were applied and only SNPs were filtered. The combined VCF file from GATK was converted to FASTA format file using a customized script made available by the Broad Institute (https://github.com/broadinstitute/broad-fungalgroup/tree/master/scripts/SNPs). Genome wide variable sites with maximum ambiguity in 10% of the total sample were concatenated. A distance matrix and a neighbor joining tree were constructed using MEGA7. Raw reads and a genome assembly from this study were submitted to NCBI under BioProject PRJNA779095.

### Metagenomic analysis of grains

For each grain reads set, human reads were initially filtered out by mapping to human genome using Kraken 2 [20] and MiniKraken2_v2_8GB database (RefSeq bacteria, archaea, and viral libraries and the GRCh38 human genome). All non-human reads were then provided with a taxonomic classification using (i) BLAST against NCBI non-redundant protein sequence database (NRDB, version 5, downloaded April 2019) and (ii) BLAST against a custom database containing *M*. *mycetomatis* assemblies (27 in-house assemblies, the mm55 genome assembly [21], and three *M*. *mycetomatis* assemblies from Khidir et al [22] and a fungal release of the UNITE database (doi: https://doi.org/10.15156/BIO/786368; [23]). Reads were assigned to *M*. *mycetomatis* if they were ≥75% identical to a *M*. *mycetomatis* database sequence over ≥75% of the alignment length. Reads that did not pass the two thresholds were further interrogated using the results of the BLAST against NRDB, using custom scripts https://github.com/appliedbinf/mycetomatis_analysis_workflow/blob/main/getNrdbGIs.pl

Reads that were ≥75% identical to an NRDB sequence over ≥75% of the alignment length were processed through a series of filters to provide them with a taxonomic classification: (i) Two sequences were considered from the same species if they were ≥95% identical over ≥95% of the alignment length. (ii) If the previous was false, the two sequences were considered from the same genus if they were ≥90% identical over ≥90% of the alignment length. (iii) If the previous was false, the two sequences were considered from the same family if they were ≥85% identical over ≥85% of the alignment length. (iv) Finally, if the previous was false, the two sequences were considered from the same order if they were ≥75% identical over ≥75% of the alignment length. Taxonomic classifications for each grain set were summarized using custom Perl scripts https://github.com/appliedbinf/mycetomatis_analysis_workflow/blob/main/getRankedTaxonomy.pl Custom scripts used for processing metagenomic data are available from github.com at https://github.com/appliedbinf/mycetomatis_analysis_workflow.

## Results

### Isolates and patient demographics

Of the 50 isolates submitted for analysis, 26 were viable and consistent with the morphology of *M*. *mycetomatis*. These isolates were from 26 different patients diagnosed with eumycetoma at the Mycetoma Research Center (MRC), University of Khartoum, Khartoum, Sudan, who were selected based on availability and represented patient population typically seen at MRC (Table 1): 20 patients had lesions on one or both feet, two had lesions on legs and knees, two had lesions on the buttocks and for two patients, the infection site was not recorded. Twenty (77%) of patients were male; median age was 35 years (range 8 to 75 y.o.)

**Table 1:** Patient demographic characteristics.

Isolate ID	Age		Sex	Occupation	Residence	Site
**Mmyc_Sud5** * ^**#**^	21	Male		Student	El Gaziera	Both feet
**Mmyc_Sud27** *	25	Male		Worker	El Gaziera	Lt foot
**Mmyc_Sud15**	29	Male		Student	El Gaziera	Lt foot
**Mmyc_Sud2**	18	Male		Jobless	El Gaziera	Rt foot
**Mmyc_Sud37**	35	Female		Farmer	Khartoum	Both feet
**Mmyc_Sud31**	16	Male		Student	White Nile	Lt foot
**Mmyc_Sud20**	8	Male		Farmer	White Nile	Lt foot
**Mmyc_Sud10**	60	Male		Farmer	El Gaziera	Rt foot
**Mmyc_Sud39**	32	Male		Worker	El Gaziera	Rt foot
**Mmyc_Sud32**	39	Female		Housewife	El Gaziera	Lt buttock
**Mmyc_Sud26**	26	Male		Employed	El Gaziera	Rt foot
**Mmyc_Sud22**	50	Female		Housewife	El Gaziera	Lt foot
**Mmyc_Sud30**		Male		NA	El Gaziera	Lt foot
**Mmyc_Sud35**	17	Male		Student	El Gaziera	Lt foot
**Mmyc_Sud16**	19	Female		Farmer	El Gaziera	Rt leg & knee
**Mmyc_Sud4** *	60	Male		Farmer	North Kordofan	Rt leg & knee
**Mmyc_Sud25** *	22	Male		Worker	North Kordofan	Rt foot
**Mmyc_Sud6**	35	Female		Farmer	El Gaziera	Lt foot
**Mmyc_Sud17**	45	Male		Worker	Sennar	Rt buttock
**Mmyc_Sud9**	54	Female		Housewife	El Gaziera	Rt foot
**Mmyc_Sud8**	39	Male		Farmer	Sennar	Lt foot
**Mmyc_Sud29**	60	Male		Farmer	Gedaref	Lt foot
**Mmyc_Sud33**	NA	Male		NA	NA	NA
**Mmyc_Sud18**	35	Male		Worker	White Nile	Both feet
**Mmyc_Sud34**	75	Male		Farmer	North Darfur	Rt foot
**Mmyc_Sud36**	NA	Male		NA	NA	NA

* isolates were from patients residing in the same village

#—reference isolate

**Fig 1:**
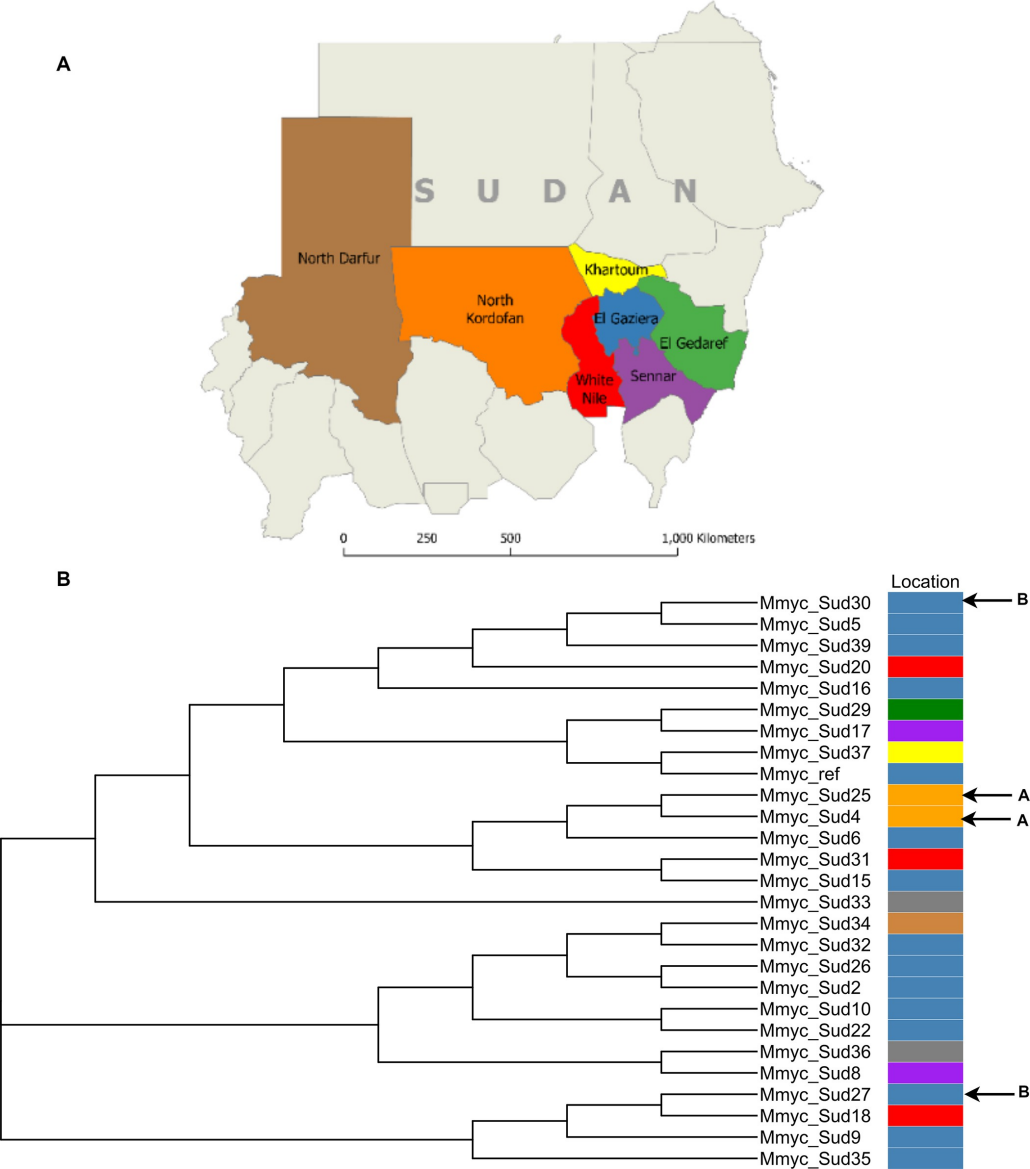
**A.** States of origin of mycetoma patients in this study. Map outline was reprinted from GADM database under a CC BY license, with permission from Global Administrative Areas (www.gadm.org), original copyright 2018. **B.** Phylogenetic relationship among isolates of *M. mycetomatis* visualized my maximum likelihood method, the colors correspond to states on the map. Isolates from patients residing in village A or B are marked with arrows. The phylogenetic tree is not shown in scale.

Most patients were from southeastern Sudan: 14 (56%) were from the State of El Gaziera, three from White Nile, two from Sennar, one from Khartoum, and one from El Gedaref. In addition, one patient was from North Darfur, two from North Kordofan and two did not report residence (Fig 1). Reported occupations included farmer, worker, student, housewife; one patient was jobless. Three patients did not report occupation (Table 1).

### Whole genome sequencing of isolates

To facilitate genomic analysis and improve existing genome assemblies of *M*. *mycetomatis* [21,22], Mmyc_Sud5 isolate was sequenced using the PacBio platform: 602,734 long reads (N50 = 25,963, Subread N50 = 10,172) were obtained resulting in over 217X depth coverage of the genome. The reads were assembled into 5 contigs 2.9–10.1 Mb in length (N50 = 9.4 Mb; Table 2). Estimated genome size was 35.7 Mb.

**Table 2:** Draft assembly statistics.

	PacBio Assembly
No of Contigs	5
N75 (bp)	9, 141,291
N50 (bp)	9, 411,943
Maximum contig length (bp)	10,135,305
Minimum contig length (bp)	2,855,995
Total length (bp)	35,695,337

The remaining isolates were sequenced using the Illumina HiSeq or MiSeq platforms. The genome coverage ranged from 99XX to 220X; for each isolate, >80% of reads were mapped covering on average 91% of the reference genome. Phylogenetic analysis using neighbor-joining and maximum likelihood methods revealed the presence of at least 7 genetically isolated clades (Fig 1). However, most of the isolates within the clades were highly clonal: pairwise differences among isolates within the same clades were 0–63 SNP; nearly identical isolates from patients residing in different states. Limited evidence of geographic structure was observed: two isolates from patients residing in village A in North Kordofan, Mmyc_Sud25 and Mmyc_Sud4 clustered together within a clade; however, the third isolate in this clade was from a patient from El Gaziera State located over 1,000 km north of North Kordofan. Two other isolates from patients residing in village B in El Gaziera, Mmyc_Sud5 and Mmyc_Sud27, were from different clades (Fig 1).

### Metagenomic analysis of DNA from grains

DNA from 81 patients diagnosed with eumycetoma at MRC was used for direct sequencing. Across all grain samples, a total of 1.3 billion Illumina HiSeq reads were obtained, of which 99.3% were of human origin (Tables 3 and S1).

**Table 3:** Read classifications from metagenomic analysis of grains.

* *	Read Count
Total	1,329,266,450
Human	1,319,835,903
*Madurella* sp.	916,681
Other (including Bacterial)	174,588

**Fig 2:**
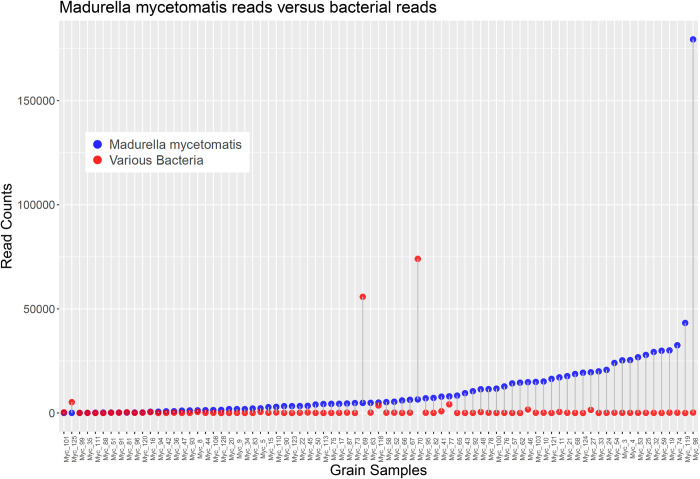
Counts of *M*. *mycetomatis*-related reads versus all combined bacterial clade reads. Grain samples are plotted along the x-axis in order of increasing *M*. *mycetomatis* content. Greater abundance of bacterial reads versus *M*. *mycetomatis* reads is apparent for the grain samples, Myc_125, Myc_69, and Myc_70.

Only 0.069% of reads were classified as related to *Madurella* sp., yet this small proportion comprised 916,681 reads. Out of the additional reads from other organisms (174,588 in Table 3), 156,816 reads originated from bacteria (Tables 3 and S2). *Madurella* sp. reads were identified in all 81 samples ranging from 51 to 179,399 reads per sample (Fig 2).

**Fig 3:**
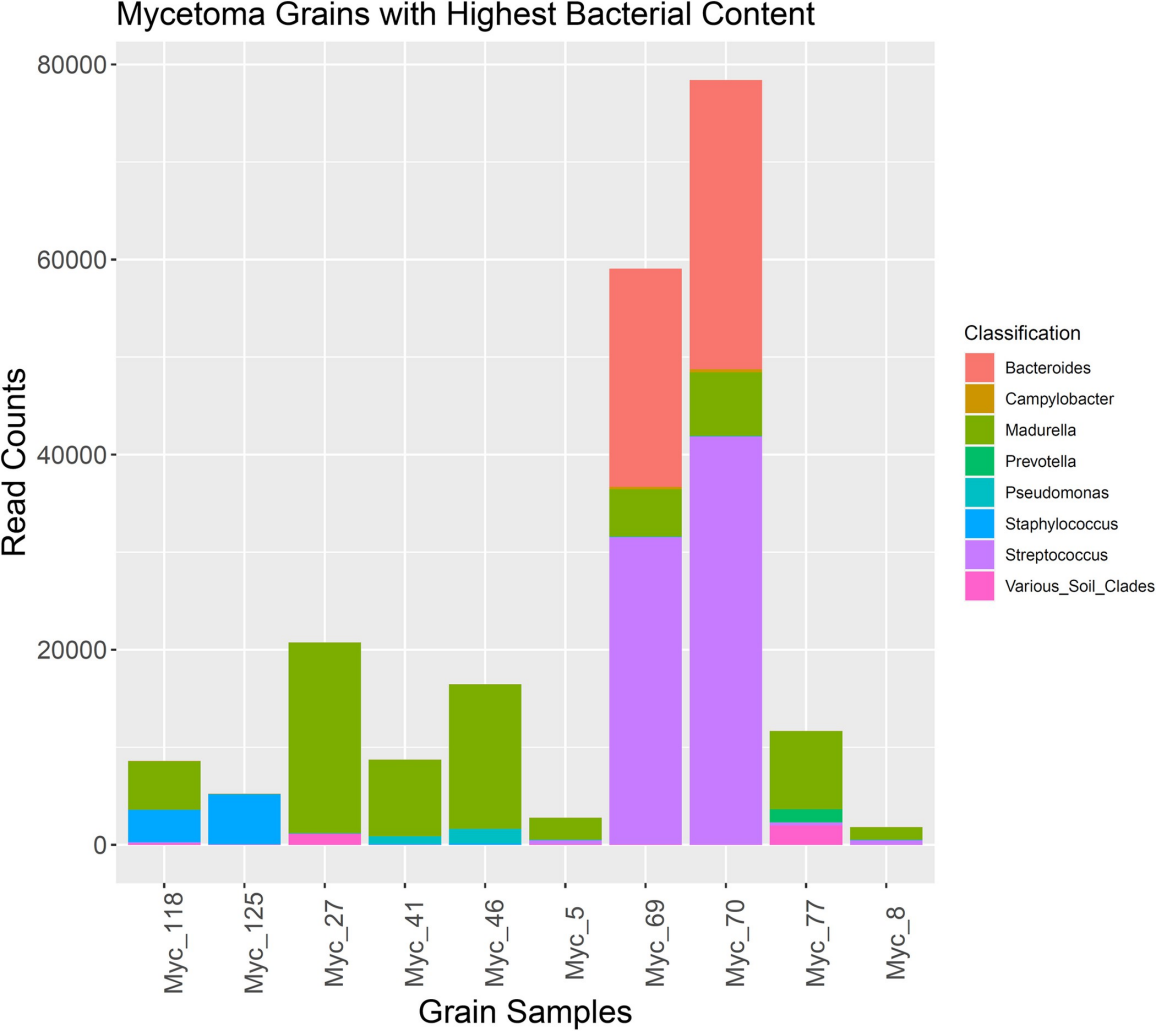
Mycetoma grain samples with top 10 highest bacterial count. In grain samples, Myc_125, Myc_69, and Myc_70, bacterial reads exceeded *M*. *mycetomatis* reads, while the latter predominated in the other seven samples shown. Bacterial reads are subdivided into genera where ‘Various soil clades’ include Rhodospirillales, Rhizobiales, and Actinobacteria.

Only four grain samples showed a predominance of bacteria (Fig 3 and S2 Table). In samples Myc_70 and Myc_69, *Streptococcus* sp. and *Bacteroides* sp. dominated while *Staphylococcus* sp. predominated in Myc_125 (Fig 3).

For sample Myc_101, with the minimum of 51 *Madurella*-related reads, 327 reads could be classified as bacterial, belonging to the soil clades Streptomycetacea and Rhizobiales. For three samples in which *Madurella* sp. comprised a majority of non-human reads, the potential pathogens, *Staphylococcus* sp. (Myc_118), *Pseudomonas* sp. (Myc_46), and *Prevotella* sp. (Myc_77), nonetheless contributed in excess of 1,000 reads each (Fig 3).

## Discussion

Better understanding of the genetic structure and diversity of mycetoma causative agents is essential for the development of diagnostic methods and identification of potential drug targets. Whole genome sequencing (WGS) provides a useful tool for this purpose as it provides numerous genetic markers that can be mined for developing diagnostic methods and identifying potential drug markers [24–26]. Here, we describe a high-quality genomic assembly of *M*. *mycetomatis*, describe genomic diversity of this species in Sudan and perform detailed metagenomic analysis of mycetoma grains from Sudanese patients.

Genomic assembly of *M*. *mycetomatis* obtained from the long-read sequencing data, consisting of 5 contigs and provided a considerable improvement over the previous assemblies that included over 800 contigs (N50 82 kB) [21,22]. Although the identification of telomeric sequences was beyond the scope of our investigation, these contigs likely represent 5 chromosomes of this fungus. This assembly will provide a framework for the future studies and the development of markers for diagnostics development.

WGS analysis of the 26 isolates of *M*. *mycetomatis* from Sudan revealed an unusual population structure of this fungus in the region. Seven genetically isolated clades differentiated by tens of thousands of SNPs were identified indicating considerable diversity in the population. At the same time, isolates within these clades from patients residing in different states were nearly genetically identical (e.g. different by 0–6 SNPs), which is lower than the within-clade diversity among isolates of the emerging pathogen, *Candida auris*, known for clonality among strains [27,28]. Further studies are needed to understand the underlying causes of clonality among strains. Inclusion of variable regions as well as indels in phylogenetic analysis may allow better differentiation between closely related isolates. Diversity among *M*. *mycetomatis* isolates has been described by others using variable-number-tandem repeat (VNTR) [29] and short-tandem-repeat (Mmy STR) assays [30]. Notably, an older study from 2003 by Ahmed et al using large scale random amplification of polymorphic DNA from *M*. *mycetomatis* isolates identified identical patterns and reported a high extent of clonality among isolates from Sudan, while observing considerable diversity among isolates of other species [31]. These results are consistent with our observations.

Direct sequencing of DNA extracted from mycetoma grains indicated that these grains primarily consist of human cells, as 99% of reads were classified as human. However, *Madurella* sp. reads were identified in all tested samples, which was consistent with the initial diagnosis of eumycetoma in these patients. However, because of the differences in the numbers of fungal cells and the amount of *Madurella* sp. DNA among grains, the numbers of *Madurella* sp. reads varied from patient to patient. Bacterial reads were identified in all 81 of the samples; however, 10 samples contained considerable numbers of the bacterial reads. Specifically, two samples, Myc_69 and Myc_70 had more *Streptococcus* spp. and *Bacteroides* spp. reads than *Madurella* sp. reads. Two other samples, Myc_118 and Myc_125 were dominated by *Staphylococcus* sp. reads. Since no clinical data were available for this group of patients, the relevance of the detection of bacterial DNA in the grains is unclear: it may indicate secondary bacterial infection or contamination from skin flora. No reads from other fungal pathogens were detected in sufficient quantities to suggest other fungal cause of mycetoma.

A recent study by Santona *et al* used an amplicon-based sequencing approach targeting an internal transcribed region (ITS1) of rDNA and identified *Madurella* sp. and *Falciformispora* sp. as the predominant fungal genera in DNA extracted from mycetoma grains; however, these authors also detected DNA of numerous other fungal genera in their samples [14]. The apparent differences between the Santona et al findings and our results are not surprising, since these authors used a PCR-based method capable of detecting small quantities of DNA that may be introduced into mycetoma lesions from soil and other outside environments. Our method that does not rely on the DNA amplification showed presence of only trace amounts of DNA of other fungal genera, which is suggestive of contamination rather than infection.

Our study was limited to the analysis of isolates and grain samples from a convenience sample of patients treated at the Mycetoma Research Center in Khartoum, Sudan; therefore, the patients’ demographic characteristics might not be representative of all mycetoma patients. Outside this region, the diversity of eumycetoma causative agents is poorly understood [3,32]. The ideal diagnostic methods for mycetoma should be able to detect and identify different causative agents of this disease to provide definitive diagnostics. Our study identified 7 genetically diverse clades in *M*. *mycetomatis* population in Sudan, other clades as well as other fungal species may be occurring in other geographic areas. Genomic studies of these pathogens from other areas are needed to develop a comprehensive understanding of the mycetoma agents for diagnostics development. Overall, our results are consistent with those by others and indicate that DNA from grains can be used as a target for molecular diagnostics; however, fungal DNA represents a relatively small proportion of the overall DNA extracted from these samples, therefore amplification is likely needed to reliably detect the presence of the pathogen in these samples.

## Supporting information

S1 Table Sample read statistics and taxonomic assignment. (XLSX)

S2 Table Microbial read counts and taxonomic assignment. (XLSX)
